# Contact with Nature and Children's Restorative Experiences: An Eye to the Future

**DOI:** 10.3389/fpsyg.2016.01885

**Published:** 2016-11-29

**Authors:** Silvia Collado, Henk Staats

**Affiliations:** ^1^Department of Psychology and Sociology, University of ZaragozaTeruel, Spain; ^2^Department of Social and Organizational Psychology, Leiden UniversityLeiden, Netherlands

**Keywords:** mental fatigue, stress recovery, understimulation, interdependencies, experience of nature, constrained restoration

## Abstract

This article offers an overview of what has been done until now on restorative research with children and opens up new inquires for future research. Most of the work has studied children's exposure to nature and the restorative benefits this contact provides, focusing on the renewal of children's psychological resources. The paper begins with an introduction to children's current tendency toward an alienation from the natural world and sets out the objectives of the article. It is followed by four main sections. The first two sections report on what we already know in this research area, distinguishing between children with normal mental capabilities and those suffering from attention-deficit hyperactivity disorder (ADHD). The findings gathered in these sections suggest that children's contact with nature improves their mood and their cognitive functioning, increases their social interactions and reduces ADHD symptoms. The next section describes five suggestions for future research: (1) the need for considering the relational dynamics between the child and the environment in restoration research, and the concept of constrained restoration; (2) the possibility of restorative needs arising from understimulation; (3) the importance of considering children's social context for restoration; (4) the relationship between restoration and pro-social and pro-environmental behaviors; and (5) children's restorative environments other than nature. We close by making some final remarks about the importance of restoring daily depleted resources for children's healthy functioning.

## Introduction

Studies conducted within research areas as environmental psychology, public health, and outdoor recreation suggest that exposure to nature can alleviate some of the negative symptoms of our children's contemporary lifestyle. Time spent in green outdoors reduces children's probability of being overweight (Cleland et al., 2010), promotes a feeling of being away from daily routines and increases relaxation (Korpela, 2002), improves children's mood (Bagot et al., 2015), and ability to focus (Wells, 2000), shapes their pro-environmental attitudes and behaviors (Chawla and Derr, 2012), and increases intergenerational social interactions (Faber Taylor et al., 1998). In spite of the recognition of the benefits children and youth obtain from contact with nature, the tendency in children's lifestyle is toward alienation of the natural world (Louv, 2008; Chawla and Derr, 2012; Myers, 2012). Both urban and rural children's independent mobility has diminished greatly in the past few years and has resulted in spending the majority of time indoors (Mattsson, 2001). Is contact with nature as essential for children as the studies above suggest?

The main goal of this article is, based on what is currently known, to outline a number of issues for future research on the benefits children obtain through direct and visual exposure to nature. In this endeavor we focus on benefits that are restorative in character, i.e., lead to the renewal or recovery of adaptive resources that have become depleted in meeting the demands of everyday life (cf. Hartig, 2004). The resources we refer to are mainly psychological (e.g., the ability to concentrate on tasks, inhibit impulses, and regain a positive mood; for a description of the two main theories of psychological restoration see Ulrich, 1983; Kaplan, 1995; Staats, 2012). We are also aware of the literature showing a positive association between children's access to nature and physical activity both in residential and school settings (e.g., Evans et al., 2012). However, we do not know whether the driving force for being more physically active is children's need for restoration. We do suggest a relationship though, to be elaborated as part of the research program we propose. Overall, we concentrate on the psychological benefits of exposure to nature.

When referring to children, we mean those from early childhood up to 18 years of age. The pattern emerging from the findings gathered until now suggests that children benefit from nature exposure as much as adults do (Wells and Rollings, 2012; Collado et al., 2016a). Little is known, however, about what specific elements and person-environment transactions make the environment restorative for children. Our departure point is an overview of research findings in this area, distinguishing between children with normal mental capabilities and those suffering from attention-deficit hyperactivity disorder (ADHD). Then, we address what we consider to be more urgent in terms of future research. The article has been organized in four main sections describing (a) restorative experiences among children with normal attention capabilities in nearby environments, (b) restoration in children suffering from ADHD, (c) specific concepts of interest and future research, and (d) final remarks.

## Restorative experiences among children with normal mental capabilities in nearby environments

Most of the work on children's restorative experiences involves settings where children spend most of their free waking hours: residential areas and school playgrounds.

### Residential settings

Residential settings constitute a restorative environment for many people, and they have attracted substantial attention in the scientific community (Hartig, 2012a; Wells and Rollings, 2012). There is evidence suggesting that exposure to nature in areas near the home improves children's psychological health, increases children's cognitive functioning (Wells, 2000), including their capacity to inhibit impulses (Faber Taylor et al., 2002) and ability to cope with stressful events (Wells and Evans, 2003). In a study with a longitudinal design Wells (2000) measured 7–12 year-olds' cognitive capabilities from low-income families before and after being relocated to a neighborhood with greater accessibility to nature. After controlling for possible confounding factors (e.g., children's pre-move cognitive functioning score, overall house quality), Wells (2000) concluded that children's cognitive functioning improved after relocation due to the higher amount of vegetation available. Faber Taylor et al. (2002) found that 7–12 year old girls with greener views from the home showed higher self-discipline, measured as their capacity to focus, to inhibit impulses and to delay gratification, compared to girls with barren views. In line with these results, Flouri et al. (2014) found access to green areas within the neighborhood to be linked to better behavioral adjustment and emotional resilience as well as to fewer problems with peers and hyperactivity. In addition, living in greener neighborhoods seems to be a protective factor against daily stressful events, such as being punished (Wells and Evans, 2003). The negative effect of 7–11 year-olds' frequency of exposure to adversity was buffered by the amount of vegetation in and around their homes. This protective effect was stronger for the most vulnerable children. Moreover, for low-income children, greener neighborhood outdoor spaces play a key role in supporting children's creative play and social interaction with adults (Faber Taylor et al., 1998).

### School settings

Children spend a large amount of time in daycare settings and schools. Thus, the restorative opportunities these environments can offer are of importance to children's healthy functioning. Considering preschoolers, Mårtensson et al. (2009) conducted a 12-days long study in which 11 preschools differing in terms of their physical features (e.g., vegetation, shrubbery, proximity of play equipment to vegetation) were selected. According to their findings, preschools with vegetation close to play structures and containing large hilly outdoor areas with trees and shrubbery boosted children's ability to concentrate (also see Carrus et al., 2015). Similarly, Roe and Aspinall (2011) concluded that 11 year-olds who spent 5 h in a forest school obtained restorative benefits, registered as mood improvement, compared to when the same children spent 5 h in a conventional indoor school setting. Interestingly, the benefits were greater for children whose normal behavioral state was characterized as bad by their teachers compared to those with good behavior. An ethnographic study showed that green schoolyards are places that enhance children's resilience and stress relief (Chawla et al., 2014). Participants also appreciated the diverse affordances (Gibson, 1979) that more natural school grounds offer. More affordances (e.g., walking around) are positively linked to children's perceived restorative qualities in schoolyards (Bagot et al., 2015).

Kelz et al. (2015) also studied children's perceptions of their schoolyard's restorative qualities. The authors demonstrated that higher access to greenery due to a renovation conducted in the school increased children's perception of compatibility (i.e., match between the child's purposes, the environmental supports for the pursuit of that purpose, and the demands imposed by the environment) and fascination (i.e., the situation automatically captures one's attention) of the schoolyard. Moreover, participants reported higher psychological well-being and had lower blood pressure compared to those in the control group.

The restorative effects of nature in educational centers have also been demonstrated indoors when, for example, a green wall is installed in the classroom (Van den Berg et al., 2016) or when children enjoy a natural window view (Liu and Sullivan, 2016).

## Restoration in children suffering from ADHD

Special interest has been drawn to the effects that nature exposure may have on children who suffer from ADHD, especially in response to the call for alternative treatment of this growing body of children (Sawni, 2008). Although, little attention has been paid to the influence of the physical environment on ADHD, there is some evidence that nature exposure helps ameliorate ADHD symptoms. For example, parents and legal guardians of children diagnosed with ADHD reported a decrease in children's ADHD symptoms after activities in green areas compared to activities in non-green areas (Faber Taylor et al., 2001; Kuo and Faber Taylor, 2004; Faber Taylor and Kuo, 2011). In a field study, Van den Berg and Van den Berg (2011) observed that ADHD children performed better on a concentration task after playing in a natural setting compared to a built setting. The findings set forth above are supported by the results of a true experiment in which ADHD children were randomly assigned to walk either in a park, a neighborhood or a downtown area (Faber Taylor and Kuo, 2009). A 20-min individually guided walk in a park proved to improve ADHD children's attention performance significantly more than in the other two conditions.

## Specific concepts of interest and future research

By now we do have a modest number of studies describing how, when, where and under what circumstances children's restorative experiences occur. But certain gaps in the literature for this specific age group are evident, considering that children's preferences and use of different environments differ from those of adults (Korpela et al., 2002) and that the restorative benefits people derive from nature vary across the life course (Astell-Burt et al., 2014). Here we highlight five areas for further investigation and make a brief methodological suggestion.

*First*, restoration research should take a closer look at the relational dynamics between the child and the environment. In particular, attention should be paid to circumstances limiting restoration, as expressed in the idea of constrained restoration (Hartig, 2012b; Von Lindern, 2015). For instance, some children's restorative needs may actually *arise* in environments generally considered to be restorative, such as natural environments. This might constrain the restorative effects that spending free time in this kind of settings has for them, as compared to children whose relationship with the same environment is merely recreational. Following this line of thought, Collado et al. (2016b) found that rural children who help their parents in their agricultural business report experiencing less restoration after spending free time in the same agricultural areas than children in the same villages whose only relationship with agricultural areas is recreational. This effect was partially due to a lower sense of being away experienced by the first group of children.

A theory helpful to understand and further develop this phenomenon of constrained restoration is the behavior setting theory (Barker, 1968). The concept of behavior setting (BS) refers to a certain pattern of behavior that is socially established in a setting, integrating the physical, psychological, and social characteristics of the environment. According to this theory, BS's can be distinguished by one to seven characteristics that determine interdependence between two BS's (for more details see Von Lindern, 2015). One of these seven is the degree of spatial interdependence, which implies that the same physical environment is used for different purposes. According to attention restoration theory (Kaplan, 1995), for restoration to occur, the individual should have a sense of being away from situations that evoke restorative needs. If the same environment is used both for restorative purposes and for activities creating restoration needs, the individual's sense of being away may be diminished and hence, his restorative experience will be constrained.

Apart from spatial interdependency, children's restorative experiences are likely to be constrained by several, quite unknown, factors (Collado et al., 2016c). From studies with adults we know that individual time constraints (Hartig et al., 2013a), cold weather conditions that may keep people inside (Hartig et al., 2013b), perceived danger (Staats and Hartig, 2004; Herzog and Rector, 2009), and heavy traffic disturbances (Von Lindern et al., 2016) are factors on which the restorative potential of a certain environment depends. Little attention has been paid, however, to the inherently relational nature of restorative environments when assessing children's restorative experiences. We encourage researchers to consider personal and sociocultural factors, such as familiarity with the environment, amount of time spent outdoors, independent mobility, compulsory activities conducted in nature (e.g., environmental education programs) and developmental stage in order to deepen our understanding of children's restorative environments.

*Second*, an issue that deserves more attention in restorative research in general, and with children in particular, is the possibility of restorative needs arising due to understimulation. As pointed out in classic studies (see Bexton et al., 1954; Hebb, 1955), human beings require varied sensory input in order to maintain adaptive behavior. We tend to look for stimulation that enhances our curiosity, risks or even fear, and experiencing these can improve individuals' resilience and coping strategies (Suedfeld, 2012). Children in natural environments seem to be keen on taking risks and challenges. This helps them acquire competence, increases their self-esteem and resilience strategies, and makes them more independent (Myers, 2012; Chawla et al., 2014). And yet we do not know whether this can be considered a restorative experience.

Nature experiences afford a varied range of activities and stimuli difficult to find in structured, indoor contexts (Chawla and Derr, 2012). Moreover, *having nothing to do* is one of the most stressful events described by children (Lewis et al., 1984). We know the restorative potential children perceive in a certain place is positively related to the affordances of the place (Bagot et al., 2015), and that exposure to nature enhances children's physical activity (Evans et al., 2012). Thus, attention should also be paid to the possible detrimental impact of understimulation for children due to their alienation from nature and the impact it has on their restorative needs and restoration opportunities. Can we demonstrate that understimulation is linked to a lack of contact with nature through experimental studies? Can we artificially stimulate children through technology, for instance through the use of gaming software that is so popular nowadays? And would understimulated children show more restorative needs than those who receive proper stimulation through contact with nature? These and other questions open up a future line of inquiry that will help us get a more in-depth understanding of children's restorative experiences.

*Third*, we should take a closer look at the social context of children's restoration. When young children are allowed to spend free time in nature, they usually engage in more cooperative and creative forms of play (Myers, 2012), describe these experiences as relaxing, and appreciate the company of friends (Chawla et al., 2014). The social meaning of restorative environments may shift across the life course. When safety is ensured, adults prefer to be alone in environments thought to support restoration, maybe trying to avoid social feedback (Staats, 2012). For children however, social contact in safe environments appears to be a key factor leading them to perceive a setting as restorative (Bagot et al., 2015). Whether this link between social interaction and restoration shifts through the life course and in what way is unknown and deserves further exploration.

These social interactions can also be analyzed from the approach of behavior setting theory. On the one hand, considering the *leadership interdependence hypothesis* (i.e., the same person takes the lead despite situations being different), one could think that being under close parental surveillance while being involved in non-organized activities in nature might constrain children's restorative experiences (Collado et al., 2016c). On the other hand, adults' guidance and support might be essential when it comes to unfamiliar environments. Moreover, parental own beliefs about the restorative potential of certain environments may shape their children's perceptions. Undoubtedly, the study of the social context of children's restorative experiences will shed some light on the way restoration works for young populations.

*Fourth*, an issue that requires further research is the relationship between children's restorative experiences and pro-environmental and pro-social behaviors. As Chawla and Derr (2012) point out, positive experiences in nature during childhood promote pro-environmentalism in children that last into adulthood. One reason for this might be children's experience of restoration within the natural environment (Collado and Corraliza, 2015). Considering the urgent need for environmental protection, a more in depth examination of the restorative processes leading children to behave in a pro-environmental way is certainly needed, preferably through longitudinal studies. Similarly, exposure to nature enhances cooperative behavior (Weinstein et al., 2009; Chawla et al., 2014) and decreases problem behavior, such as bullying (Matsuoka, 2010). However, the pathways leading to pro-sociability through contact with nature are fairly unknown. Being able to restore depleted resources might be one of the reasons for the nature exposure-pro-sociability association.

*Fifth*, children's favorite places are associated with their experiences of restoration (e.g., feelings of relaxation; Korpela, 2002). This does not imply, however, that their favorite place is a natural one (Korpela et al., 2002), nor that the main factor leading to children's restoration is exposure to nature (Bagot et al., 2015). The study of children's restorative environments other than nature is worthwhile, and a close examination of their favorite places might be a good start.

*Finally*, if our aim is to gain a deeper understanding of children's restorative experiences, new methodologies need to be employed. First, an effort should be made to conduct more true or quasi-experimental studies, so that we can disentangle the *unique* effect of nature from other confounding variables. This would also rule out the possibility that more restored children choose to spend more time in nature and not the other way around. Nevertheless, considering that the results obtained in correlational studies are in line with the theoretical background as well as the findings of quasi-experimental research (e.g., Wells, 2000; Faber Taylor and Kuo, 2009), we are pretty confident that it is exposure to nature that enhances restorative outcomes. Second, researchers should register *nature* in a more sophisticated way, combining objective data of the physical environment with people's perceptions (Collado et al., 2016d). The general use of the term *nature* should make way to one that is more specific with what we mean by nature exposure in terms of quantity and quality of nature, as well as length of this exposure. Third, quantitative data should be combined with interviews, observations and other qualitative measures likely to provide a more comprehensive understanding of how the process of restoration works for children.

## Final remarks

The material presented in this paper suggests that children's access to nature offers opportunities for restoration and contributes to their everyday functioning. Although, in some respects the findings gathered until now parallel results with adults, the uniqueness of children's restorative experiences should be considered and translated in research that takes this directly into account. This includes the aspects mentioned in the previous section, with a special emphasis on evaluating children's restorative environments by focusing on their functions, rather than their forms (Heft, 2012). In addition, we need to be more creative in our methodological approach, adapting our research practices to young populations. Given the importance of psychological restoration for children's functioning, there is also an urgent need for a closer collaboration between researchers, city planners, educators and parents in order to apply insights from children's restoration research. After all, we share the final aim of guarding our children's healthy development.

## Author contributions

Conceived the ideas included in the paper: SC and HS. Wrote the paper: SC and HS.

## Funding

This study was partly supported by the Spanish Ministry of Economy and Competitiveness (Grant number PSI-2013-44939).

### Conflict of interest statement

The authors declare that the research was conducted in the absence of any commercial or financial relationships that could be construed as a potential conflict of interest.
